# Sex differences in changes of depressive symptoms among older adults before and during the COVID-19 pandemic: evidence from two longitudinal cohorts

**DOI:** 10.1186/s12877-023-03744-1

**Published:** 2023-02-01

**Authors:** Fanfan Zheng, Chenglong Li, Rong Hua, Jie Liang, Darui Gao, Wuxiang Xie

**Affiliations:** 1 School of Nursing, Peking Union Medical College, Chinese Academy of Medical Sciences, Shijingshan District, 33 Ba Da Chu Road, Beijing, 100144 China; 2 Peking University Clinical Research Institute, Peking University First Hospital, Haidian District, No. 38 Xueyuan Road, Beijing, 100191 China

**Keywords:** Sex differences, Depressive symptoms, COVID-19, Older adults, Longitudinal study

## Abstract

**Background:**

Major concerns about the adverse mental health impact of the rapidly spread COVID-19 pandemic have been raised. Previous studies on changes of depressive symptoms during the COVID-19 pandemic have yielded inconsistent results regarding the sex differences. Since women have higher depressive symptoms even without the pandemic, it is essential to consider the pre-existing change of depressive symptoms of a similar period to discern the effect of the pandemic on depression. This study aimed to evaluate sex differences in depressive symptoms before and during the pandemic.

**Methods:**

Data from the Health and Retirement Study (HRS; waves 13 to 15) and the English Longitudinal Study of Ageing (ELSA; wave 8 to COVID-19 wave 2) were analyzed. Depressive symptoms were assessed by the 8-item Center for Epidemiological Studies Depression (CES-D) scale. According to the time of COVID-19 outbreak in the US and the UK, the intervals from waves 13 to 14 surveys of the HRS and from waves 8 to 9 surveys of the ELSA were employed as pre-pandemic periods to control for the pre-existing depressive symptoms, respectively. Changes of CES-D scores during the pre-pandemic and pandemic periods were assessed by linear mixed models.

**Results:**

Nine thousand, seven hundred thirty-seven participants (mean age: 66.7 ± 10.7 years) from the HRS and 5,098 participants (mean age: 68.7 ± 10.0 years) from the ELSA were included. CES-D scores among women were significantly higher than those among men at all waves in both cohorts. During the pre-pandemic period, no significant sex difference on changes of CES-D scores was detected in either the HRS or the ELSA. During the pandemic period, CES-D scores were increased in both men and women and the sex differences in CES-D increments of the two cohorts were both significant. Enlarged sex differences were demonstrated in increments of CES-D scores during the pandemic period.

**Conclusions:**

Our results suggest women suffered from worse depressive symptoms in response to the pandemic, although the changes of depression were similar between men and women before the pandemic. These findings underscore the necessity to support the vulnerable populations, especially women, to manage the distress brought by the pandemic and maintain optimal mental health status.

**Supplementary Information:**

The online version contains supplementary material available at 10.1186/s12877-023-03744-1.

## Background

The COVID-19 pandemic, caused by severe acute respiratory syndrome coronavirus 2, has disrupted people’s daily life profoundly and unprecedentedly from every aspect. The concerns of mental health have emerged soon after the spread of the pandemic, as the scale of changes in mental health, as well as information of the most vulnerable individuals, is of great importance to policy makers and service providers. One major mental health problem, which has attracted increasing attention, is depression. Previous studies investigating the change of depressive symptoms have yielded mixed results, with some reporting similar levels of depressive symptoms before and during the pandemic [1], while others reporting significant increase in depressive symptoms during the pandemic [2–4]. Apart from that, a growing number of recent studies have investigated sex differences in depressive symptoms and inconsistent findings were observed [1, 2, 5, 6]. Those discrepancies might due to the use of convenience samples, differences in measures of depressive symptoms, and most importantly, inadequate baseline data from pre-pandemic period to accurately calculate the unfolding impact of the pandemic. Besides, regarding sex differences in depression, it is worth noting that, compared with men, women have higher prevalence and incidence of depression during their lifetime even without the COVID-19 pandemic [7, 8]. Thus, to precisely capture the influence of the pandemic on depressive symptoms in different gender groups, it is necessary to measure the pre-pandemic depressive symptoms to determine whether previous finding of sex differences in depressive symptoms was just a reflection of pre-existing sex differences or truly induced by the pandemic.

To tackle this caveat, assessments of depressive symptoms from several time points before and during the pandemic are critical to disentangle the effect directly induced by the pandemic and compare the changes of depressive symptoms during different periods. Large and nationally representative populations with multiple assessments of depressive symptoms before and during the pandemic, such as the Health and Retirement Study (HRS), which was comprised of individuals over age 50 in the USA [9], and the English Longitudinal Study of Ageing (ELSA), which was designed as a sister study to the HRS and consisted of community-dwelling individuals aged ≥ 50 years in England [10], meet this requirement and therefore provide a golden opportunity to explore the changes of depressive symptoms before and during the pandemic, especially in relation to sex differences. By using data from the HRS and the ELSA, the present study was aiming to determine: 1) whether sex differences existed in pandemic-induced depressive symptoms; and 2) whether the magnitude of sex differences in pandemic-induced depressive symptoms was larger than that of pre-pandemic period.

## Methods

### Study design and participants

We designed a longitudinal cohort study derived from the HRS and the ELSA, which have collected health data before and during COVID-19 pandemic. Both the HRS and the ELSA are prospective, ongoing, biennial, and nationally representative cohorts among adults aged over 50 years to understand all aspects of ageing in the US and the UK, respectively. Detailed cohort profiles including objectives, designs, participants, and methods of both cohorts have been published [9–11].

**Fig. 1:**
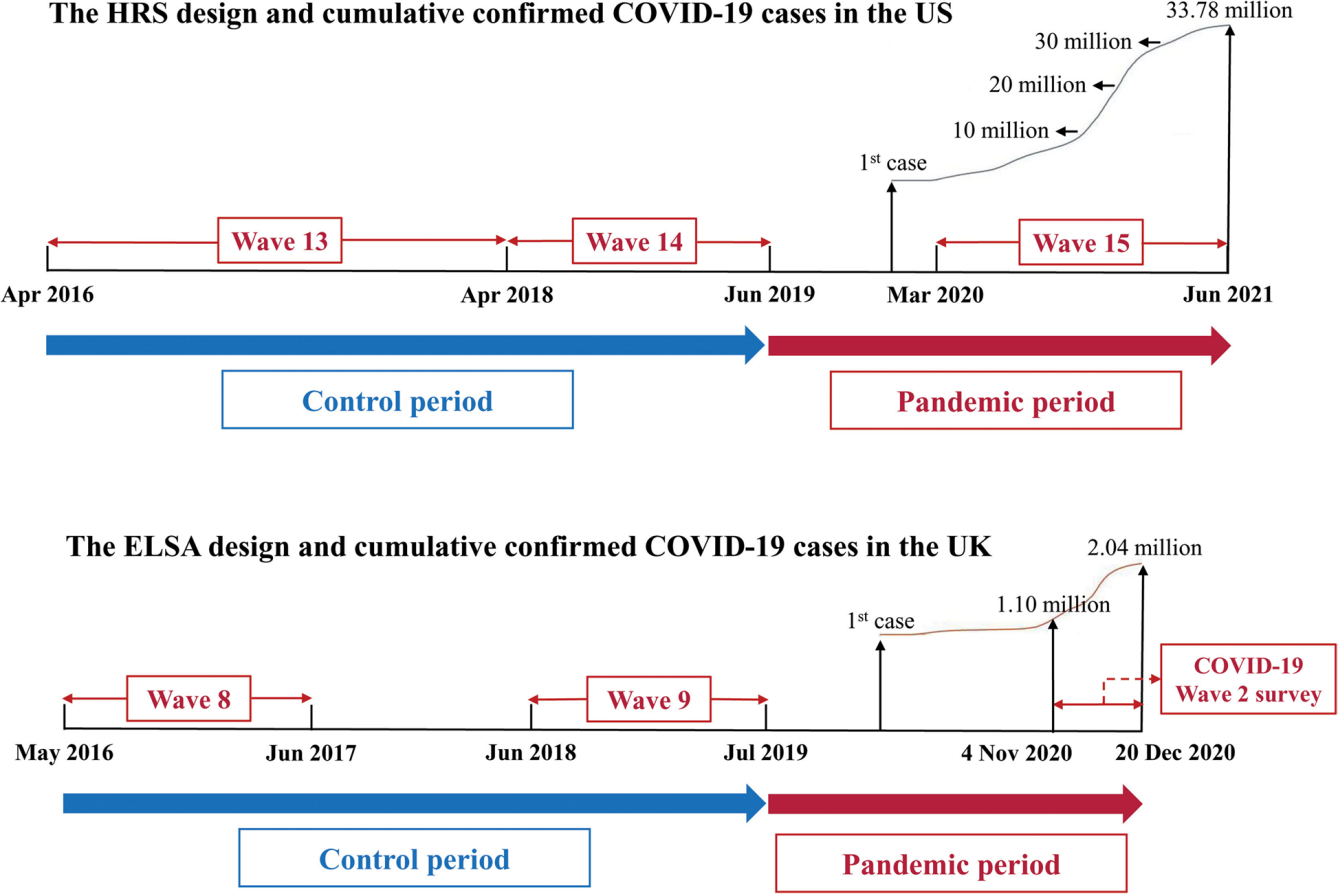
Study design and timeline of the HRS and the ELSA, and cumulative confirmed COVID-19 cases in the US and the UK. HRS = Health and Retirement Study; ELSA = English Longitudinal Study of Ageing

As shown in Fig. 1, the interval from wave 13 (2016 to 2018) to wave 14 (2018 to 2019) surveys of the HRS was defined as control period before COVID-19 pandemic, and the interval between wave 14 and wave 15 (March 2020 to June 2021) was defined as the period during COVID-19 pandemic. Similarly, the intervals of wave 8 (2016 to 2017) to wave 9 (2018 to 2019), and wave 9 to COVID-19 wave 2 survey (November 4 to December 20, 2020) of the ELSA were defined as control period and COVID-19 pandemic period, respectively.

Wave 14 of the HRS and wave 9 of the ELSA were considered as the baseline surveys. As presented in Figure S1 (Additional file 1), of 17,146 participants attending the wave 14 survey of the HRS, 2,941 participants were excluded from our analyses due to missing data on depressive symptoms measured by using 8-item Center for Epidemiological Studies Depression (CES-D) scale at any one of the three waves from 13 to 15, and additional 4,468 participants with doctor-diagnosed depression before COVID-19 pandemic were excluded. In the ELSA, 8,736 participants responded to the survey of wave 9, and 3,638 of them were excluded because of incomplete data on CES-D scores measured at waves 8 to 9 and COVID-19 wave 2 survey, or doctor-diagnosed depression before the pandemic (Figure S2 in Additional file 1). Finally, 9,737 participants from the HRS and 5,098 from the ELSA were included this study.

### Pandemic period and pre-pandemic period

The first confirmed case of COVID-19 found in the US was reported on January 20, 2020 [12]. During the fieldwork period of the HRS wave 15 survey from March 1, 2020 to June 30, 2021, the cumulative confirmed cases in the US increased from 32 to 33.78 million (Fig. 1). On January 27, 2020, the first two cases were diagnosed in the UK [13]. During COVID-19 wave 2 survey of the ELSA, from November 4 to December 20, 2020, the cumulative confirmed cases increased from 1.10 million to 2.04 million in the UK (Fig. 1). Both cohort studies added COVID-19-related questions to the latest interviews, and most interviews were conducted via telephone or web due to the restrictions on social contact during the fieldwork periods.

Baseline characteristics of participants in both cohorts included age, race (white or non-white), education (≥ 12 or < 12 years), cohabitation status (living alone was defined as never married, legally separated, divorced or widowed), current smoking (yes or no), drinking (at least once per week), moderate and vigorous physical active (at least once per week), and self-reported doctor-diagnosed hypertension, diabetes, heart disease, stroke, and cancer.

### Outcomes

The primary outcome was the changes of CES-D scores during pre-pandemic periods (control period) and during the pandemic period. Both cohorts used 8-item CES-D scale to measure the depressive symptoms of participants. This version of the CES-D scare has an internal consistency and factor structure that are comparable with longer versions of the scale [14]. The CES-D scores were the sum of six negative items and two positive items. The negative items measured whether the participant experienced the following sentiments all or most of the time in the past week: depression, everything is an effort, sleep is restless, felt alone, felt sad, and could not get going (answering yes was scored 1 and no was scored 0). The positive items measured whether the respondent felt happy and enjoyed life, all or most of the time in the past week (answering no was scored 1 and yes was scored 0). The CES-D scores ranged from 0 to 8, with higher scores meaning a great severity of depressive symptoms. According to previous studies, depressive symptoms were defined as CES-D scores ≥ 4 [15, 16].

### Statistical analysis

Two cohorts were analyzed independently, while the pooled analysis was not performed as high heterogeneity was observed between two cohorts. Mean ± standard deviation (SD) and n (%) were used to describe continuous and categorical variables, respectively. Baseline characteristic differences between sexes were tested by using t test or chi-square test for continuous and categorical variables, respectively.

Linear mixed models were developed to evaluate the sex differences in changes of CES-D scores during pre-pandemic period and during pandemic period after adjusting for baseline age, race, education, cohabitation status, current smoking, drinking, moderate and vigorous physical active (at least once per week), and self-reported doctor-diagnosed hypertension, diabetes, heart disease, stroke, and cancer. Sex and time were considered as classified variables in linear mixed models. Time = 1 indicates wave 13 of the HRS or wave 8 of the ELSA; time = 2 indicates wave 14 of the HRS or wave 9 of the ELSA; time 3 indicates wave 15 of the HRS or COVID-19 wave 2 of the ELSA. Firstly, least squares means (LSMs) and 95% confidence intervals (CIs) of CES-D scores by sex and time were evaluated by models. Secondly, LSM differences in CES-D scores between sexes at each wave were calculated, and then the sex differences in the changes of CES-D scores during pre-pandemic period and during pandemic period were estimated. Finally, we used the sex differences in the changes of CES-D scores during pre-pandemic period as the references, and investigated whether the sex differences in the changes of CES-D scores during COVID-19 pandemic period were significantly higher.

Besides, modified Poisson regression models were used to analyze the association between sex and incident depressive symptoms during pandemic period among participants without depressive symptoms before the pandemic after adjusting for baseline CES-D scores and those covariates mentioned above [17]. Relative risks (RR) and 95% CIs were estimated to present the strength of association between sex and incident depressive symptoms.

Several sensitivity analyses were performed to evaluate the stability of our main results in different subgroups. First, we repeated our main analyses using linear mixed models in participants without depressive symptoms before the pandemic. Second, we explored whether there were significant modified effects from covariates and COVID-19 infection on the sex differences in the changes of CES-D scores during pandemic period compared with those during control period.

All analyses were performed by using SAS 9.4 (SAS Institute Inc.), and a 2-sided alpha value of 0.05 was considered statistically significant.

## Results

### Baseline characteristics

Nine thousand, seven hundred thirty-seven participants (4,312 men and 5,425 women; mean age: 66.7 ± 10.7 years) from the HRS and 5,098 participants (2,227 men and 2,871 women; mean age: 68.7 ± 10.0 years) from the ELSA were included this study. Table 1 presents baseline characteristics of individuals by sex. In both cohorts, women had lower percentages of drinking, moderate and vigorous physical active, hypertension, diabetes, and heart disease, had a higher percentage of living alone and depressive symptoms, and had higher CES-D scores than men.

**Table 1:** Characteristics of individuals at baseline

Characteristics	HRS (wave 14, *n* = 9737)			ELSA (wave 9, *n* = 5098)		
	**Men (*n* = 4312)**	**Women (*n* = 5425)**	*p* for difference*	**Men (*n* = 2227)**	**Women (*n* = 2871)**	*p* for difference*
Age (years)	66.7 ± 10.3	66.7 ± 11.1	0.952	69.1 ± 9.8	68.3 ± 10.2	0.004
White (%)	2910 (67.5)	3509 (64.7)	0.004	2169 (97.4)	2782 (96.9)	0.294
Education ≥ 12 years (%)	3711 (86.1)	4726 (87.1)	0.129	816 (36.6)	877 (30.6)	< 0.001
Living alone (%)	974 (22.6)	2338 (43.1)	< 0.001	528 (23.7)	1043 (36.3)	< 0.001
Current smoking (%)	495 (11.5)	486 (9.0)	< 0.001	176 (7.9)	214 (7.5)	0.550
Drinking ≥ once per week (%)	1045 (24.2)	768 (14.2)	< 0.001	1479 (66.4)	1389 (48.4)	< 0.001
Moderate and vigorous physical active (%)	3363 (78.0)	3906 (72.0)	< 0.001	1758 (78.9)	2135 (74.4)	< 0.001
Hypertension (%)	2612 (60.6)	3099 (57.1)	< 0.001	791 (35.5)	884 (30.8)	< 0.001
Diabetes (%)	1188 (27.6)	1299 (23.9)	< 0.001	247 (11.1)	193 (6.7)	< 0.001
Heart disease (%)	1059 (24.6)	924 (17.0)	< 0.001	149 (6.7)	84 (2.9)	< 0.001
Stroke (%)	291 (6.8)	311 (5.7)	0.039	47 (2.1)	47 (1.6)	0.213
Cancer (%)	626 (14.5)	757 (14.0)	0.429	109 (4.9)	129 (4.5)	0.501
CES-D scores	0.86 ± 1.36	1.01 ± 1.52	< 0.001	0.94 ± 1.46	1.39 ± 1.79	< 0.001
Depressive symptoms (%)	249 (5.8)	402 (7.4)	0.001	142 (6.4)	356 (12.4)	< 0.001

Data were presented as mean ± SD or n (%)

*HRS* Health and Retirement Study, *ELSA* English Longitudinal Study of Ageing, *CES-D* Center for Epidemiological Studies Depression

^*^P value reported for differences between sexes using t test or chi-square test

### Sex differences in changes of CES-D scores during different periods

As shown in Tables 2 and 3, CES-D scores in women were significantly higher than those in men at all waves in both cohorts. However, no significant changes were observed in CES-D scores during pre-pandemic period among both sexes in the HRS, and the LSM difference between sexes in the changes of CES-D scores was also not significant (-0.04, 95% CI: -0.09 to 0.02, *p* = 0.220). In the ELSA (Table 3), although CES-D scores significantly increased from wave 8 to wave 9 in both sexes, sex difference in the increments of CES-D scores was not significant (-0.01, 95% CI: -0.10 to 0.08, *p* = 0.801).

**Table 2:** Sex differences in the changes of CES-D scores in the HRS

	**CES-D scores, LSM (95% CI)**^**a**^			
	**Men (*n* = 4312)**	**Women (*n* = 5425)**	**LSM differences between sexes**^**a**^	***p* for differences between sexes**^**a**^
**Before COVID-19 pandemic**				
Wave 13 (2016)	0.86 (0.82 to 0.91)	0.99 (0.95 to 1.03)	0.12 (0.07 to 0.18)	< 0.001
Wave 14 (2018)	0.89 (0.85 to 0.94)	0.98 (0.91 to 1.02)	0.09 (0.03 to 0.15)	0.003
LSM differences between waves^a^	0.03 (-0.01 to 0.07)	-0.01 (-0.05 to 0.03)	-0.04 (-0.09 to 0.02)	0.220
*P* for differences between waves^a^	0.174	0.751	0.220	/
**During COVID-19 pandemic**				
Wave 14 (2018)	0.89 (0.85 to 0.94)	0.98 (0.91 to 1.02)	0.09 (0.03 to 0.15)	0.003
Wave 15 (2020)	0.98 (0.94 to 1.03)	1.15 (1.11 to 1.19)	0.17 (0.11 to 0.24)	< 0.001
LSM differences between waves^a^	0.09 (0.05 to 0.14)	0.17 (0.13 to 0.21)	0.08 (0.02 to 0.14)	0.024
*P* for differences between waves^a^	< 0.001	< 0.001	0.024	/
**During COVID-19 pandemic vs. Before COVID-19 pandemic**				
Differences in LSM differences between two periods^a^	0.06 (-0.01 to 0.14)	0.18 (0.11 to 0.24)	0.12 (0.01 to 0.21)	0.037
*P* for differences in LSM differences between two periods^a^	0.077	< 0.001	0.037	/

Data on sex differences in the changes of CES-D scores before and during the COVID-19 pandemic in the HRS were presented

*HRS* Health and Retirement Study, *CES-D* Center for Epidemiological Studies Depression, *LSM* Least Squares Mean

^a^after adjusting for age, race, education, cohabitation status, current smoking, alcohol consumption, exercise, hypertension, diabetes, heart disease, stroke, and cancer at wave 14, by using linear mixed models

**Table 3:** Sex differences in the changes of CES-D scores in the ELSA

	**CES-D scores, LSM (95% CI)**^**a**^			
	**Men (*n* = 2227)**	**Women (*n* = 2871)**	**LSM differences between sexes**^**a**^	***p* for differences between sexes**^**a**^
**Before COVID-19 pandemic**				
Wave 8 (2016)	0.92 (0.85 to 0.99)	1.27 (1.21 to 1.32)	0.34 (0.26 to 0.43)	< 0.001
Wave 9 (2018)	1.01 (0.94 to 1.07)	1.34 (1.28 to 1.40)	0.33 (0.24 to 0.42)	< 0.001
LSM differences between waves^a^	0.09 (0.02 to 0.15)	0.07 (0.02 to 0.13)	-0.01 (-0.10 to 0.08)	0.801
*P* for differences between waves^a^	0.010	0.011	0.801	/
**During COVID-19 pandemic**				
Wave 9 (2018)	1.01 (0.94 to 1.07)	1.34 (1.28 to 1.40)	0.33 (0.24 to 0.42)	< 0.001
COVID-19 Wave 2 (2020)	1.69 (1.59 to 1.78)	2.32 (2.24 to 2.40)	0.63 (0.51 to 0.76)	< 0.001
LSM differences between waves^a^	0.68 (0.59 to 0.77)	0.98 (0.90 to 1.05)	0.30 (0.18 to 0.41)	< 0.001
*P* for differences between waves^a^	< 0.001	< 0.001	< 0.001	/
**During COVID-19 pandemic vs. Before COVID-19 pandemic**				
Differences in LSM differences between two periods^a^	0.59 (0.47 to 0.72)	0.91 (0.79 to 1.01)	0.32 (0.14 to 0.48)	< 0.001
*P* for differences in LSM differences between two periods^a^	< 0.001	< 0.001	< 0.001	/

Data on sex differences in the changes of CES-D scores before and during the COVID-19 pandemic in the ELSA were presented

*ELSA* English Longitudinal Study of Ageing, *CES-D* Center for Epidemiological Studies Depression, *LSM* Least Squares Mean

^**a**^after adjusting for age, race, education, cohabitation status, current smoking, alcohol consumption, exercise, hypertension, diabetes, heart disease, stroke, and cancer at wave 9, by using linear mixed models

During COVID-19 pandemic periods, significant increments of CES-D scores were observed among men and women in both cohorts (Tables 2 and 3). In the HRS, the changes in CES-D scores from wave 14 to wave 15 were 0.09 (95% CI: 0.05 to 0.14, *p* < 0 0.001) and 0.17 (95% CI: 0.13 to 0.21, *p* < 0.001) in men and women, respectively, and the sex difference was 0.08 (95% CI: 0.02 to 0.14, *p* = 0.024) using men as the reference. Compared with the results from the HRS, the increments of CES-D scores during the pandemic period in the ELSA were significantly higher: 0.68 (95% CI: 0.59 to 0.77, *p* < 0.001) in men and 0.98 (95% CI: 0.90 to 1.05, *p* < 0.001) in women, respectively (Table 3). The sex difference in these two increments was also significant (0.30, 95% CI: 0.18 to 0.41, *p* < 0.001).

Compared with increments of CES-D scores during pre-pandemic periods, significantly higher increments were observed during pandemic periods among women in both cohorts: 0.18 (95% CI: 0.11 to 0.24, *p* < 0.001) and 0.91 (95% CI: 0.79 to 1.01, *p* < 0.001) in the HRS and the ELSA, respectively. However, such significantly higher increment among men, during COVID-19 pandemic vs. before COVID-19 pandemic period, was only observed in the ELSA (0.59, 95% CI: 0.47 to 0.72, *p* < 0.001). In addition, we used sex differences in the increments of CES-D scores during pre-pandemic periods as the references, and found that sex differences in increments during pandemic period were significantly higher in both cohorts: 0.12 (95% CI: 0.01 to 0.21, *p* = 0.037) and 0.32 (95% CI: 0.14 to 0.48, *p* < 0.001) in the HRS and the ELSA, respectively (Tables 2 and 3).

As shown in Table 4, the crude incidences of incident depressive symptoms among women were significantly higher than those among men without depressive symptoms before COVID-19 pandemic in both cohorts. Although the crude incidences were significantly different between two cohorts, the strengths of association between sex and incident depressive symptoms in the HRS and the ELSA were similar after adjusting for baseline CES-D scores and other covariates (RR = 1.40, 95% CI: 1.16 to 1.69 in the HRS, and 1.42, 95% CI: 1.23 to 1.64 in the ELSA, respectively, using men as the reference).

**Table 4:** Association between sex and new-onset depressive symptoms during COVID-19 pandemic

	Crude incidence, n (%)*		**Relative risk (95% CI)**^**a**^	
	**The HRS**	**The ELSA**	**The HRS**	**The ELSA**
Men	168 (4.3)	255 (12.7)	Reference	Reference
Women	302 (6.3)	488 (20.8)	1.40 (1.16 to 1.69)	1.42 (1.23 to 1.64)
*P* for differences between sexes	< 0.001	< 0.001	< 0.001	< 0.001

Data on association between sex and new-onset depressive symptoms during COVID-19 pandemic among participants without depressive symptoms before COVID-19 pandemic were presented

*HRS* Health and Retirement Study, *ELSA* English Longitudinal Study of Ageing, *CES-D* Center for Epidemiological Studies Depression

^**a**^after adjusting for age, race, education, cohabitation status, current smoking, alcohol consumption, exercise, hypertension, diabetes, heart disease, stroke, cancer, and CES-D scores at baseline (wave 14 of the HRS and wave 9 of the ELSA), by using modified Poisson regression models

^*^P value reported for differences between sexes using chi-square test

### Sensitivity analyses

Several sensitivity analyses have been performed. Firstly, main analyses using linear mixed models were repeatedly conducted among participants without depressive symptoms before COVID-19 pandemic in both cohorts. As shown in Tables S1 and S2 (Additional file 1), the changes in CES-D scores during pre-pandemic periods were similar, while the changes during pandemic periods became greater in both cohorts. In addition, sex differences in increments of CES-D scores during COVID-19 pandemic vs. before COVID-19 pandemic periods also became greater. Secondly, subgroup analyses have been performed to explore modified effects on the sex differences. However, no significant modified effect was observed in both cohorts (Figures S3 and S4 in Additional file 1).

## Discussion

By using data from two large, nationally representative cohorts of older adults in the US and the UK, we observed significant sex differences in pandemic-induced depressive symptoms, with women demonstrating a greater increase in depressive symptoms than men do during the COVID-19 pandemic. During the pre-pandemic period, no significant sex difference on changes of CES-D scores was detected in either the HRS or the ELSA. After accounting for pre-existing depressive symptoms, we found significant sex differences in CES-D increments during the pandemic period in both cohorts. The extent of sex differences in changes of depressive symptoms during the pandemic was significantly larger than that of pre-pandemic period.

To the best of our knowledge, this is the first study employing a pre-pandemic period as a control period to detect whether sex differences existed in additional depressive symptoms induced by the pandemic among older adults. Sex differences in depressive symptoms and related disorders are among the most intriguing mental health issues and has attracting more and more attention after the pandemic started due to its significant clinical and public health importance. Previous studies with regard to this question have reported discordant results. Several studies found remarkably higher depressive symptoms in women or girls [1, 2, 4, 18–20], while others revealed similar levels of depressive symptoms between different gender groups [5, 21–24]. These mixed findings might be explained, at least in part, by differences in study designs, populations, and measurements of depressive symptoms. Given discrepancies regarding the mentioned aspects above, making comparisons with prior studies seems impossible. Since it is well-documented that depression is more common among women than among men even without the pandemic [7, 8], it is necessary to take pre-pandemic depression level into account to identify whether sex differences existed in pandemic-induced depression. Although a few studies with longitudinal designs have considered the level of pre-pandemic depressive symptoms, they only accounted for the pre-existing depressive symptoms of a single time point [1, 2]. As depressive symptoms tend to fluctuate over time [25, 26], the depressive symptoms of a single time cannot fully represent the level of pre-pandemic depressive symptoms, leading to potential bias to subsequent results. Thus, our study adds to previous research with the delicate design by using a similar period before the pandemic as control to account for pre-pandemic depressive symptoms. Interestingly, although women showed higher depression in all waves of both cohorts, no significant sex difference in changes of depressive symptoms during pre-pandemic period was detected. Further analyses demonstrated significant sex differences in additional depressive symptoms induced by the pandemic, and the magnitude of change in depressive symptoms during the pandemic was much higher than that of pre-pandemic period, resulting in an even larger extent of sex differences.

Another interesting finding is, although we found significant sex differences in both cohorts, it seems like the US individuals were more resilient than the UK populations in face of the COVID-19 pandemic, as the range of increase in CES-D scores of the HRS was significantly lower than that of the ELSA. However, this may have something to do with the apparently more strict policy in the UK. Owing to the rapid spread of COVID-19, the UK government has urged people to stay at home and issued the forced isolation policy (also known as lockdown) for several times. The COVID-19 wave 2 survey of the ELSA was happened from November 4 to December 20, 2020, which was almost simultaneous with the second national lockdown, which came into force in England on 5 November, 2020 and ended after 4 weeks at 2 December, 2020. Therefore, it is highly possible that the higher depressive symptoms in the ELSA were attributable to the imposed lockdown, which did not only amplify the fear of getting infected, but also bring challenges from fulfilling basic needs of food, water, medications, and safe accommodation to further problems related to the financial aspect such as cuts in income, losses of employment, and inability to pay bills [27]. In fact, previous studies have already reported that people at the early stages of the lockdown showed elevated levels of depression [6], and the mental health situations deteriorated during the lockdown when compared with that of pre-lockdown [28]. Intriguingly, being a woman is a risk factor for higher level of depression or worse mental health status in both studies [6, 28], which is consistent with findings of the present study to a certain extent. These data, together with our findings, may suggest the importance of supporting individuals, especially those at high risk for mental health issues, in particular women, to reduce negative affects during implement of future policy in face of the COVID-19 pandemic. Despite this, it is worth mentioning that, although the crude incidences of depressive symptoms were higher in the ELSA than in the HRS, the strengths of association of sex with incident depressive symptoms in the HRS and the ELSA were very similar, revealing a relatively stable effect of sex on pandemic-induced depressive symptoms.

According to previous literatures, several proposed mechanisms may link the pandemic to the observed sex differences in changes of depressive symptoms in the present study. The first potential mechanism is related to the gene-environment interaction given the COVID-19 pandemic is a very stressful environmental factor with no doubt. Genetic risk factors were supposed to partly explain sex differences in depression as the heritability of major depressive disorder is estimated to be over 30% [29]. Although the findings of a higher genetic risk for women than for men is still inconclusive [29, 30], the moderating effects of sex on severity of depressive symptoms have been reported in several genes [31–34]. For instance, the short allele of 5-*HTTLPR* was associated with increased risk for major depressive disorder in women and stressful life events could enhance the effect [35]; and female carriers of minor allele of rs1360780 in *FKBP5* displayed more depressive symptoms under high external stress [36]. Hence, the COVID-19 pandemic, as a stressful life event, may act as the external environmental factor and exert its effect differently between gender groups via gene-environment interaction, leading to more severe depressive symptoms in women. Despite this, sex hormones, with their well-established impacts on neurotransmitter systems in the brain, have been linked to the increased risk of depressive disorders in women and girls [34, 37, 38], and might play an essential role in inducing enlarged sex differences of depression during the pandemic as the stressful event may cause violent fluctuations in hormone and neurotransmitter systems. Another possible mechanism involves different hypothalamic–pituitary–adrenal (HPA) axis responses to psychosocial stress between men and women. In general, women show less HPA axis activation to stress as an evolutionary result of the fact that women need to attenuate their stress response to protect the fetus from adverse effects of psychosocial stress during maternal period [39]. Yet the blunted HPA axis response to psychosocial stress, such as the COVID-19 pandemic, could contribute to risk of depression [40]. Other possible mechanisms regarding the gender gap of depressive symptoms referred to the determinants of gender equality consisted of social status, access to resources, and economic positions, etc. [41, 42], all of which seemed to be expanded between different gender groups during the pandemic in the short term [43, 44]. Indeed, the mechanisms mentioned above may represent coinciding independent biological cascades that aggregate to exacerbate the gender gap of depressive symptoms during the COVID-19 pandemic.

### Strengths and limitations

The major strength of the present study is the application of a control period. By using data collected from a similar period before the pandemic as control, we are able to calculate the pandemic-induced depressive symptoms and measure the actual magnitude of the additional depressive symptoms. Given the fluctuated nature of depressive symptoms [25, 26], previous longitudinal studies accounted for pre-pandemic depressive symptoms of a single time may be inaccurate and lead to potential bias to the results [1, 2]. A recent research reported increased depression among older adults as the pandemic progressed by using data from three time points; however, the changes in depression was estimated by comparing the prevalence of depression of the three time points without measuring the magnitude of changes in depressive symptoms, which is represented by CES-D score [4]. Another strength of the present study is the inclusion of two nationally representative cohorts comprised of populations living in different countries with distant COVID-19 policies. The robust and consistent findings between the two cohorts remarkably enhanced the certainty and generalizability of our results. Nevertheless, our study also has several limitations and the present findings should be cautiously interpreted. First, the depressive symptoms in both cohorts were assessed by a short version of the original 20-item CES-D scale, namely the 8-item CES-D scale, which may be not sensitive enough to detect subtle changes in depressive symptoms. However, the internal consistency and factor structure of the 8-item scale (Cronbach’s alpha: 0.78) was comparable with the original scale (Cronbach’s alpha: 0.84–0.85) [14], and the threshold of 4 or more on the short version was equivalent to the conventional cutoff point of 16 or over on the original CES-D scale [45]. Second, although we adjusted for a number of potential confounders, the possibility of residual confounding, such as genetic susceptibility factors, cannot be ruled out. Third, 7,409 participants from the HRS and 3,638 from the ELSA were excluded from the study due to incomplete data or doctor-diagnosed depression. Due to the significant differences in baseline characteristics between participants included and excluded in both cohorts (Tables S3 and S4 in Additional file 1), potential selection bias might exist.

## Conclusions

The findings of this study demonstrated deteriorated depression status and enlarged sex differences in depressive symptoms during the COVID-19 pandemic, emphasizing the necessity to provide additional support to older adults, especially women, to manage or reduce the stress brought by the persistent pandemic. Future policies should take place not only in coping with the threat of infection of the COVID-19, but also in developing ways to supporting the vulnerable groups to attenuate the adverse impact on mental health and maintain optimal mental health status during the pandemic crisis.

## Supplementary Information

**Additional file 1: Figure S1.** Flow chart of participant selection for the HRS. **Figure S2.** Flow chart of participant selection for the ELSA. **Figure S3.** Subgroup analyses to identify potential modified effects from covariates and COVID-19 infection on the sex differences in the changes of CES-D scores during pandemic period compared with those during control period in the HRS. **Figure S4.** Subgroup analyses to identify potential modified effects from covariates and COVID-19 infection on the sex differences in the changes of CES-D scores during pandemic period compared with those during control period in the ELSA. **Table S1.** Sensitivity analysis in participants without depressive symptoms before COVID-19 pandemic in the HRS (waves 13 and 14): sex differences in the changes of CES-D scores before and during the COVID-19 pandemic. **Table S2.** Sensitivity analysis in participants without depressive symptoms before COVID-19 pandemic in the ELSA (waves 8 and 9): sex differences in the changes of CES-D scores before and during the COVID-19 pandemic. **Table S3.** Comparison of baseline characteristics between participants included (*n*=9,737) and excluded due to missing baseline data on CES-D scores or doctor-diagnosed depression (*n*=7,409) in the HRS. **Table S4.** Comparison of baseline characteristics between participants included (*n*=5,098) and excluded due to missing baseline data on CES-D scores (*n*=3,638) in the ELSA.

## Data Availability

The data used in this study are available from the UK Data Service (https://ukdataservice.ac.uk/) and the Health and Retirement Study (https://hrsonline.isr.umich.edu/), subject to registration and application process.

## Abbreviations

HRS: The Health and Retirement Study
ELSA: The English Longitudinal Study of Ageing
CES-D: Center for Epidemiological Studies Depression scale
