# Supplementary material for: Sex differences in changes of depressive symptoms among older adults before and during the COVID-19 pandemic: evidence from two longitudinal cohorts
Source: BMC Geriatr. 2023 Feb 1;23:64. doi: 10.1186/s12877-023-03744-1 (PMC9891753; doi:10.1186/s12877-023-03744-1)
Supplement: Supplementary file 1 — Additional file 1: Figure S1. Flow chart of participant selection for the HRS. Figure S2. Flow chart of participant selection for the ELSA. Figure S3. Subgroup analyses to identify potential modified effects from covariates and COVID-19 infection on the sex differences in the changes of CES-D scores during pandemic period compared with those during control period in the HRS. Figure S4. Subgroup analyses to identify potential modified effects from covariates and COVID-19 infection on the sex differences in the changes of CES-D scores during pandemic period compared with those during control period in the ELSA. Table S1. Sensitivity analysis in participants without depressive symptoms before COVID-19 pandemic in the HRS (waves 13 and 14): sex differences in the changes of CES-D scores before and during the COVID-19 pandemic. Table S2. Sensitivity analysis in participants without depressive symptoms before COVID-19 pandemic in the ELSA (waves 8 and 9): sex differences in the changes of CES-D scores before and during the COVID-19 pandemic. Table S3. Comparison of baseline characteristics between participants included (n=9,737) and excluded due to missing baseline data on CES-D scores or doctor-diagnosed depression (n=7,409) in the HRS. Table S4. Comparison of baseline characteristics between participants included (n=5,098) and excluded due to missing baseline data on CES-D scores (n=3,638) in the ELSA. [file 12877_2023_3744_MOESM1_ESM.pdf]

Additional file

Fanfan Zheng, Chenglong Li, Rong Hua, Jie Liang, Darui Gao, Baoliang Zhong, Wuxiang Xie. Sex differences in changes of depressive symptoms among older adults before and during the COVID-19 pandemic: evidence from two longitudinal cohorts.

Figure S1. Flow chart of participant selection for the HRS.
Figure S2. Flow chart of participant selection for the ELSA.
Figure S3. Subgroup analyses to identify potential modified effects from covariates and COVID-19 infection on the sex differences in the changes of CES-D scores during pandemic period compared with those during control period in the HRS.
Figure S4. Subgroup analyses to identify potential modified effects from covariates and COVID-19 infection on the sex differences in the changes of CES-D scores during pandemic period compared with those during control period in the ELSA.
Table S1. Sensitivity analysis in participants without depressive symptoms before COVID-19 pandemic in the HRS (waves 13 and 14): sex differences in the changes of CES-D scores before and during the COVID-19 pandemic.
Table S2. Sensitivity analysis in participants without depressive symptoms before COVID-19 pandemic in the ELSA (waves 8 and 9): sex differences in the changes of CES-D scores before and during the COVID-19 pandemic.
Table S3. Comparison of baseline characteristics between participants included (n=9,737) and excluded due to missing baseline data on CES-D scores or doctor-diagnosed depression (n=7,409) in the HRS.
Table S4. Comparison of baseline characteristics between participants included (n=5,098) and excluded due to missing baseline data on CES-D scores (n=3,638) in the ELSA.

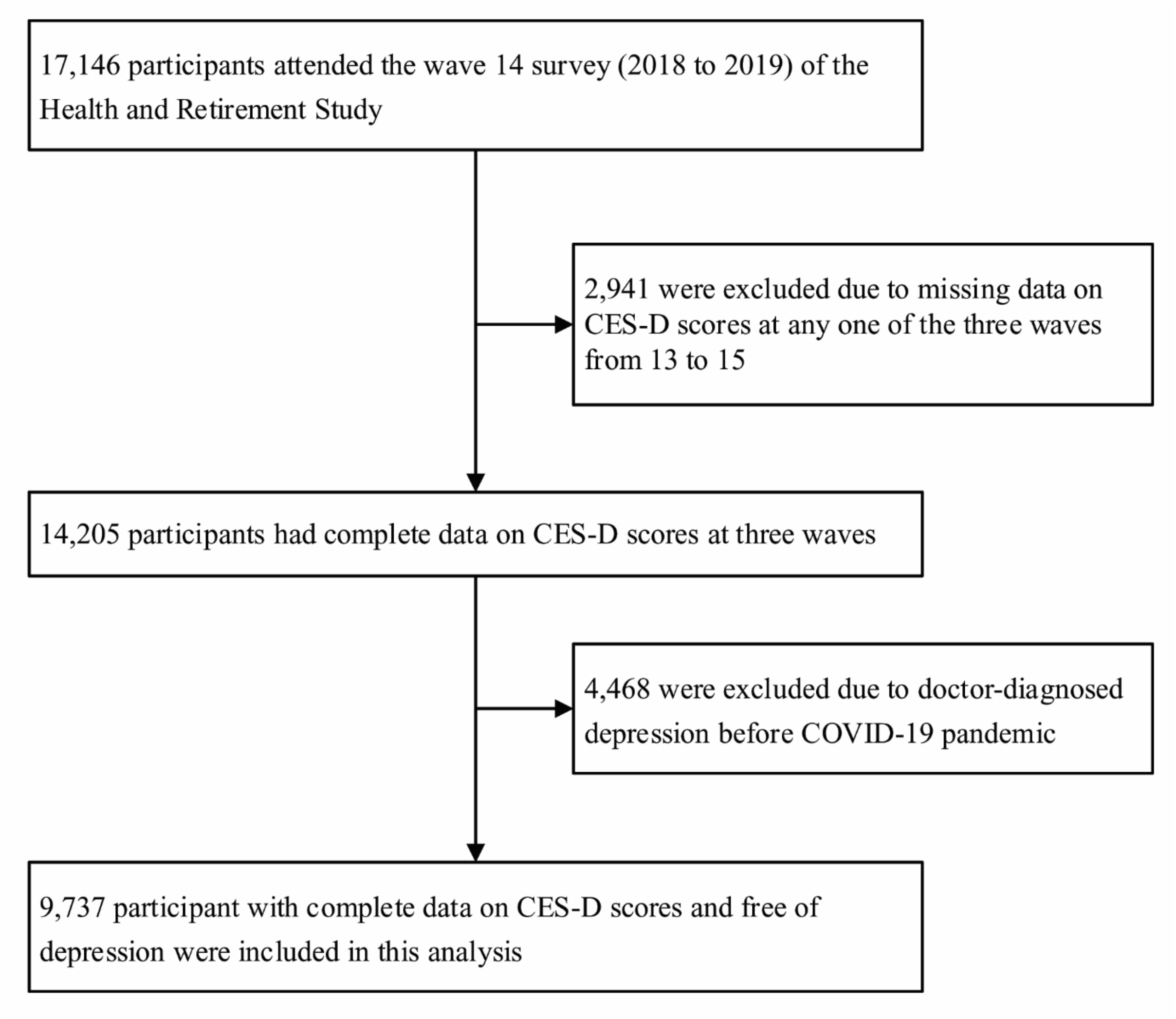

Figure S1. Flow chart of participant selection for the HRS

HRS = Health and Retirement Study; CES-D = Center for Epidemiological Studies Depression.

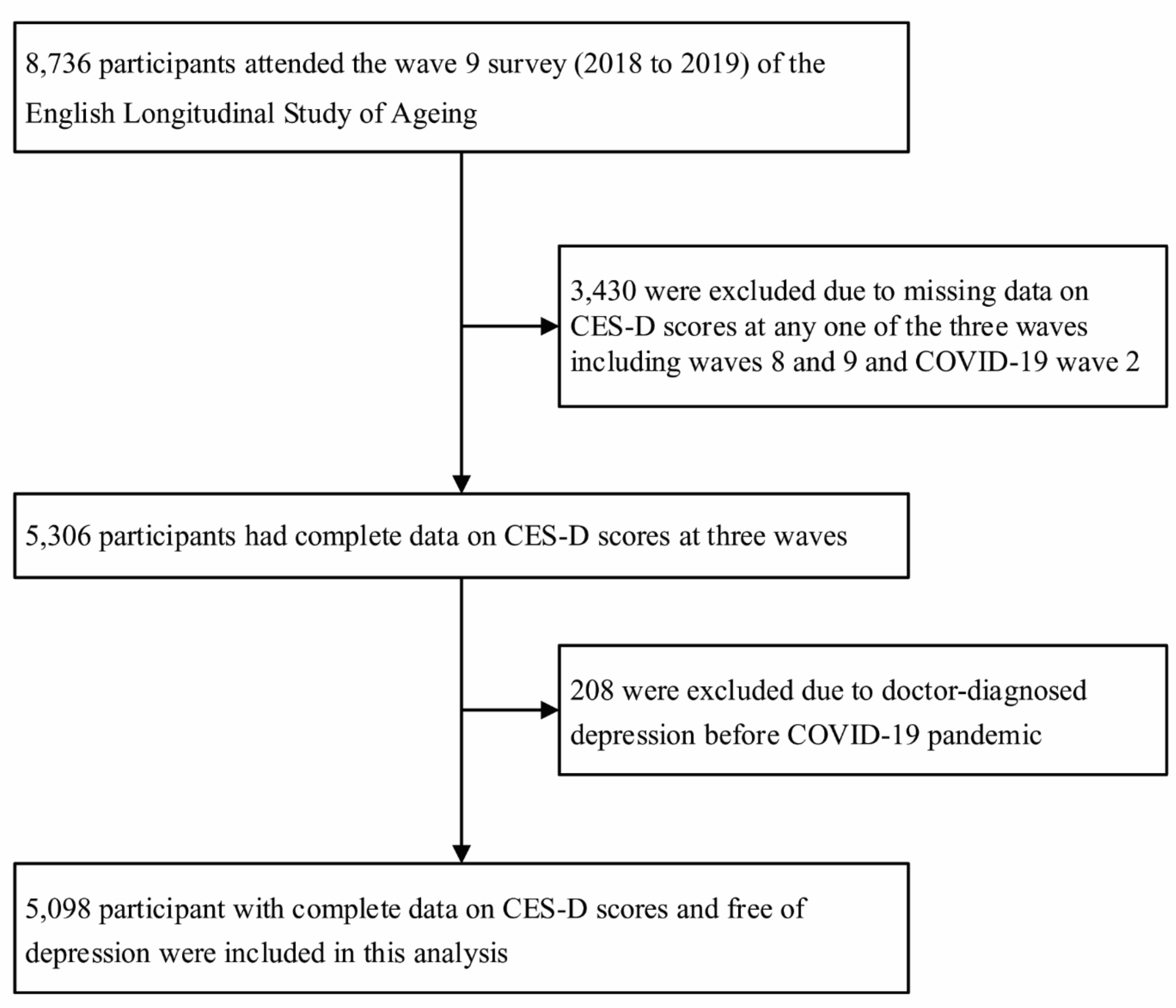

Figure S2. Flow chart of participant selection for the ELSA

ELSA = English Longitudinal Study of Ageing; CES-D = Center for Epidemiological Studies Depression.

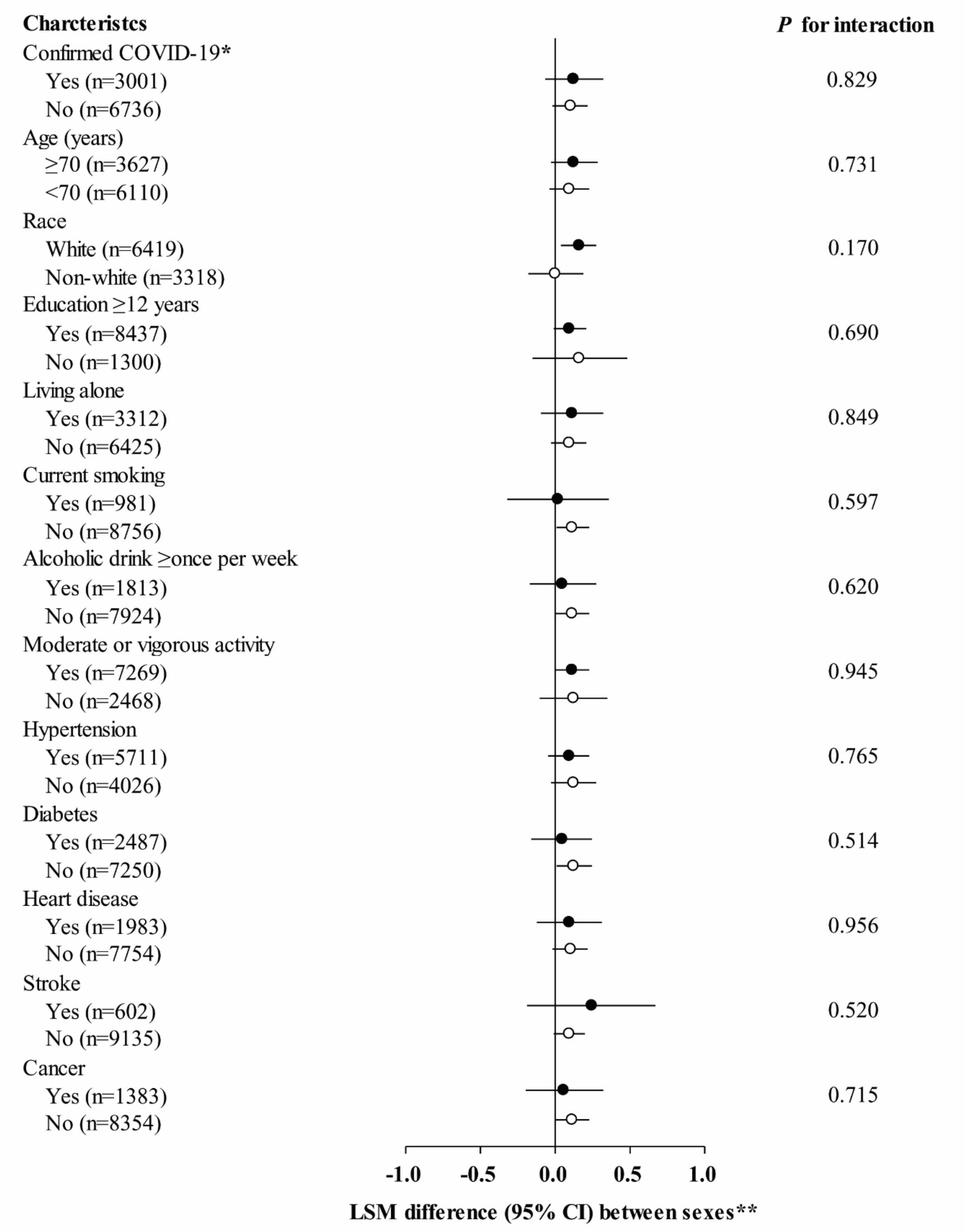

Figure S3. Subgroup analyses to identify potential modified effects from covariates and COVID-19 infection on the sex differences in the changes of CES-D scores during pandemic period compared with those during control period in the HRS

*the participant own self, a family member, a relative or a friend has been diagnosed with COVID-19.

**after adjusting for age, race, education, cohabitation status, current smoking, alcohol consumption, exercise, hypertension, diabetes, heart disease, stroke, and cancer at wave 14, except where an adjusting variable was itself being tested, by using linear mixed models.

HRS = Health and Retirement Study; CES-D = Center for Epidemiological Studies Depression; LSM = Least Squares Mean.

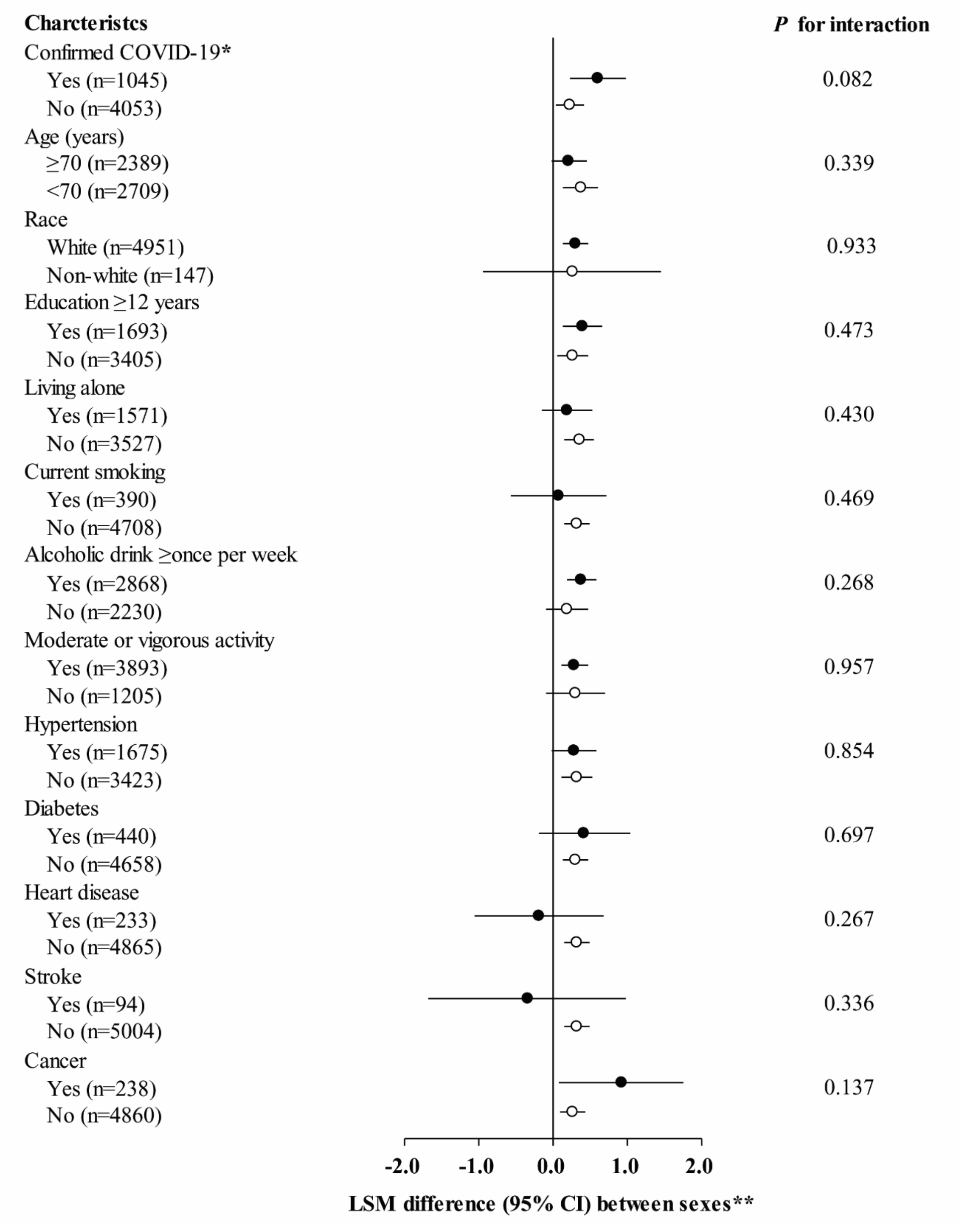

Figure S4. Subgroup analyses to identify potential modified effects from covariates and COVID-19 infection on the sex differences in the changes of CES-D scores during pandemic period compared with those during control period in the ELSA

*the participant own self, a family member, a relative or a friend has been diagnosed with COVID-19.

**after adjusting for age, race, education, cohabitation status, current smoking, alcohol consumption, exercise, hypertension, diabetes, heart disease, stroke, and cancer at wave 9, except where an adjusting variable was itself being tested, by using linear mixed models.

ELSA = English Longitudinal Study of Ageing; CES-D = Center for Epidemiological Studies Depression; LSM = Least Squares Mean.

Table S1. Sensitivity analysis in participants without depressive symptoms before COVID-19 pandemic in the HRS (waves 13 and 14): sex differences in the changes of CES-D scores before and during the COVID-19 pandemic

	CES-D scores, LSM (95% CI)*			
	**Men (n=3914)**	**Women (n=4774)**	**LSM differences between sexes***	***P* for differences between sexes***
**Before COVID-19 pandemic**				
Wave 13 (2016)	0.56 (0.54 to 0.59)	0.63 (0.61 to 0.66)	0.07 (0.03 to 0.11)	<0.001
Wave 14 (2018)	0.57 (0.55 to 0.60)	0.61 (0.59 to 0.64)	0.04 (0.01 to 0.08)	0.032
LSM differences between waves*	0.01 (-0.02 to 0.04)	-0.02 (-0.04 to 0.01)	-0.03 (-0.07 to 0.01)	0.120
P for differences between waves*	0.325	0.219	0.120	/
**During COVID-19 pandemic**				
Wave 14 (2018)	0.57 (0.55 to 0.60)	0.61 (0.59 to 0.64)	0.04 (0.01 to 0.08)	0.032
Wave 15 (2020)	0.76 (0.72 to 0.81)	0.92 (0.89 to 0.96)	0.16 (0.10 to 0.21)	<0.001
LSM differences between waves*	0.19 (0.15 to 0.23)	0.31 (0.27 to 0.34)	0.12 (0.06 to 0.17)	<0.001
P for differences between waves*	<0.001	<0.001	<0.001	/
**During COVID-19 pandemic vs. Before COVID-19 pandemic**				
Differences in LSM differences between two periods*	0.18 (0.12 to 0.23)	0.33 (0.27 to 0.38)	0.15 (0.07 to 0.23)	<0.001
P for differences in LSM differences between two periods *	<0.001	<0.001	<0.001	/

*after adjusting for age, race, education, cohabitation status, current smoking, alcohol consumption, exercise, hypertension, diabetes, heart disease, stroke, and cancer at wave 14, by using linear mixed models.

HRS = Health and Retirement Study; CES-D = Center for Epidemiological Studies Depression; LSM = Least Squares Mean.

Table S2. Sensitivity analysis in participants without depressive symptoms before COVID-19 pandemic in the ELSA (waves 8 and 9): sex differences in the changes of CES-D scores before and during the COVID-19 pandemic

	CES-D scores, LSM (95% CI)*			
	**Men (n=2007)**	**Women (n=2349)**	**LSM differences between sexes***	***P* for differences between sexes***
**Before COVID-19 pandemic**				
Wave 8 (2016)	0.54 (0.50 to 0.58)	0.73 (0.69 to 0.76)	0.19 (0.14 to 0.24)	<0.001
Wave 9 (2018)	0.62 (0.58 to 0.66)	0.77 (0.73 to 0.80)	0.15 (0.10 to 0.20)	<0.001
LSM differences between waves*	0.08 (0.03 to 0.12)	0.04 (-0.00 to 0.08)	-0.04 (-0.10 to 0.02)	0.221
P for differences between waves*	<0.001	0.052	0.221	/
**During COVID-19 pandemic**				
Wave 9 (2018)	0.62 (0.58 to 0.66)	0.77 (0.73 to 0.80)	0.15 (0.10 to 0.20)	<0.001
COVID-19 Wave 2 (2020)	1.34 (1.25 to 1.43)	1.88 (1.80 to 1.96)	0.54 (0.42 to 0.66)	<0.001
LSM differences between waves*	0.72 (0.64 to 0.81)	1.11 (1.04 to 1.19)	0.39 (0.28 to 0.51)	<0.001
P for differences between waves*	<0.001	<0.001	<0.001	/
**During COVID-19 pandemic vs. Before COVID-19 pandemic**				
Differences in LSM differences between two periods*	0.64 (0.54 to 0.75)	1.08 (0.98 to 1.17)	0.43 (0.29 to 0.57)	<0.001
P for differences in LSM differences between two periods *	<0.001	<0.001	<0.001	/

*after adjusting for age, race, education, cohabitation status, current smoking, alcohol consumption, exercise, hypertension, diabetes, heart disease, stroke, and cancer at wave 14, by using linear mixed models.

ELSA = English Longitudinal Study of Ageing; CES-D = Center for Epidemiological Studies Depression; LSM = Least Squares Mean.

Table S3. Comparison of baseline characteristics between participants included (n=9,737) and excluded due to missing baseline data on CES-D scores or doctor-diagnosed depression (n=7,409) in the HRS

Characteristic	Included (n = 9,737)	Excluded (n = 7,409)	*P* for difference*
Age (years)	66.7±10.7	67.4±12.2	<0.001
Female (%)	5425 (55.7)	4691 (63.3)	<0.001
White (%)	6419 (65.9)	4876 (65.8)	0.878
Education ≥12 years (%)	8437 (86.7)	5850 (79.0)	<0.001
Living alone (%)	3312 (34.0)	3372 (45.5)	<0.001
Current smoking (%)	981 (10.1)	1267 (17.1)	<0.001
Drinking ≥once per week (%)	1813 (18.6)	1072 (14.5)	<0.001
Moderate and vigorous physical active (%)	7269 (74.7)	4390 (59.3)	<0.001
Hypertension (%)	5711 (58.7)	5024 (67.8)	<0.001
Diabetes (%)	2487 (25.5)	2477 (33.4)	<0.001
Heart disease (%)	1983 (20.4)	2260 (30.5)	<0.001
Stroke (%)	602 (6.2)	986 (13.3)	<0.001
Cancer (%)	1383 (14.2)	1276 (17.2)	<0.001

The results are presented as mean ± SD, or n (%).

*Calculated by using a t test, or chi-square test.

ELSA = English Longitudinal Study of Ageing; CES-D = Center for Epidemiological Studies Depression.

Table S4. Comparison of baseline characteristics between participants included (n=5,098) and excluded due to missing baseline data on CES-D scores or doctor-diagnosed depression (n=3,638) in the ELSA

Characteristic	Included (n = 5,098)	Excluded (n = 3,638)	*P* for difference*
Age (years)	68.7±10.0	63.2±17.1	<0.001
Female (%)	2871 (56.3)	2011 (55.3)	0.335
White (%)	4951 (97.1)	3347 (92.0)	<0.001
Education ≥12 years (%)	1693 (33.2)	1151 (31.6)	0.122
Living alone (%)	1571 (30.8)	1431 (39.3)	<0.001
Current smoking (%)	390 (7.7)	459 (12.6)	<0.001
Drinking ≥once per week (%)	2868 (56.3)	735 (20.2)	<0.001
Moderate and vigorous physical active (%)	3893 (76.4)	2380 (65.4)	<0.001
Hypertension (%)	1675 (32.9)	776 (21.3)	<0.001
Diabetes (%)	440 (8.6)	259 (7.1)	0.010
Heart disease (%)	233 (4.6)	140 (3.9)	0.100
Stroke (%)	94 (1.8)	85 (2.3)	0.109
Cancer (%)	238 (4.7)	130 (3.6)	0.012

The results are presented as mean ± SD, or n (%).

*Calculated by using a t test, or chi-square test.

HRS = Health and Retirement Study; CES-D = Center for Epidemiological Studies Depression.
